# Roles of *SfHSP70-7* in Thermal Tolerance, Insecticide Adaptability, and Reproductive Performance Under Combined Environmental Stress of the Rice Pest *Sogatella furcifera* (Hemiptera: Delphacidae)

**DOI:** 10.3390/insects17080765

**Published:** 2026-07-25

**Authors:** Zhenzhen Wang, Yi Yan, Chongfen Yi, Hongwei Zhang, Dengquan Liu, Zhanlie Yang

**Affiliations:** 1 Guizhou Rice Research Institute, Guiyang 550006, China; wangzhenzhen714@yeah.net (Z.W.); cfyi0066@163.com (C.Y.); zhwcaas@163.com (H.Z.); 2 Guizhou Key Laboratory of Agricultural Biosecurity, College of Biological and Environmental Engineering, Guiyang University, Guiyang 550005, China; yanheyi95@163.com; 3 Plant Protection and Quarantine Station of Huangping County, Huangping 556100, China; 13885551936@163.com

**Keywords:** *Sogatella furcifera*, RNA interference (RNAi), fecundity, thermal tolerance

## Abstract

*Heat shock protein 70* (*HSP70*) regulates the stress tolerance and reproductive ability of insects. In this study, the *SfHSP70-7* gene was cloned and identified from the rice pest *Sogatella furcifera*. SfHSP70-7 encodes a typical cytoplasmic HSP70 protein that has a conserved functional domain and is highly similar to the HSP70 homologous protein of *Laodelphax striatellus*. *SfHSP70-7* was found to be widely expressed across all developmental stages, with the highest levels in nymphs and female adults. In terms of tissue distribution, it was particularly abundant in the gut and ovaries. Its expression was strongly induced by high/low temperatures and three insecticides (triflumezopyrim, sulfoxaflor, and imidacloprid), with the maximum induction under heat and triflumezopyrim stress. RNA interference (RNAi) efficiently silenced *SfHSP70-7* and significantly increased the susceptibility of *S. furcifera* to triflumezopyrim and sulfoxaflor, but it had no effect on susceptibility to imidacloprid. RNAi knockdown of *SfHSP70-7* markedly reduced survival by 45.5% (30 °C) and 38.9% (35 °C) under heat stress. In addition, gene knockdown can damage the reproductive performance of females by reducing oviposition and egg hatchability. These results indicate that *SfHSP70-7* is involved in regulating the heat tolerance, insecticide adaptability, and reproductive regulation mechanism of *S. furcifera*, providing a potential target for pest control.

## Simple Summary

The white-backed planthopper *Sogatella furcifera* is a major rice pest, and clarifying which genes regulate its stress resistance and reproduction is critical for sustainable pest control. Here, we characterized *SfHSP70-7*, a cytoplasmic HSP70 gene with conserved domains. This gene was widely expressed across developmental stages and was abundant in the gut and ovary, and its expression was strongly induced by high temperatures and insecticides. RNAi knockdown of *SfHSP70-7* lowered heat survival, increased susceptibility to two insecticides, and impaired female fecundity.

## 1. Introduction

The white-backed planthopper, *Sogatella furcifera* (Horvath), is one of the major migratory pests during the rice growth process. It is widely distributed in the rice-growing regions of Asia and Oceania, including South, Southeast, and East Asia; northern Australia; and the South Pacific islands [1]. Both its adults and nymphs feed on the phloem sap of rice plants through piercing–sucking mouthparts, causing tissue damage and nutrient loss. Moreover, it is the main transmission vector of Southern Rice Black-Streaked Dwarf Virus (SRBS-DV), which seriously affects rice yield and quality [2]. During growth, *S. furcifera* frequently encounters diverse adverse abiotic stresses, such as high/low temperatures, pesticides, and ultraviolet (UV) radiation, which influence its growth, survival, and geographical distribution [3].

Heat shock proteins (HSPs) are highly conserved molecular chaperones that play a key role in protecting insects from various environmental stresses, including extreme temperatures, pesticides, heavy metals and ultraviolet radiation [4]. Among all HSP families, HSP70 is the largest and most thoroughly studied, with members distributed in different cell compartments, including mitochondria, endoplasmic reticulum, cytoplasm and plastid matrix [5]. HSP70 members localized to different subcellular compartments perform distinct but complementary functions in insect stress responses. Cytosolic HSP70s bind misfolded proteins and prevent their aggregation, and their rapid induction through heat, cold, and insecticide stress enhances insect thermotolerance and insecticide tolerance [6]. The ER-resident member GRP78/BiP regulates the unfolded protein response and maintains ER homeostasis, while mitochondrial HSP70 (mtHSP70/GRP75) preserves mitochondrial integrity and energy metabolism under adverse conditions [7]. Together, these compartment-specific HSP70s form a coordinated chaperone network enabling insects to survive environmental challenges [8].

Global temperature increases and widespread use of pesticides are two major anthropogenic factors that exert great selection pressure on the insect population [9]. Previous studies have shown that the HSP70 gene is significantly upregulated in a variety of Hemiptera pests (*Laodelphax striatellus*, *Nilaparvata lugens*, and *Rhopalosiphum padi*) at high temperatures [9,10,11]. Similarly, insecticide exposure has been shown to induce *HSP70* expression. For instance, sublethal concentrations of lambda-cyhalothrin increase the transcript levels of *RpHSP70-1* and *RpHSP70-2* by 4–8-fold in *R. padi* [11]. In one study, treating *Myzus persicae* with LC_50_ lambda-cyhalothrin for 6–36 h can significantly induced *MpHsp70* expression, which was is 4–9 times that of the control group. After silencing the gene, the sensitivity of *M. persicae* adults to pesticides increased greatly, and the mortality rate increased by 22.6% [2].

Although previous studies have identified five *SfHsp70* genes in *S. furcifera*’s response to abiotic stress, the complete repertoire of *HSP70* genes in this species has not been fully clarified, and their functions in response to commonly used pesticides (such as trifluoropyrimidine, sulfonyl fluoride and imidacloprid) have not been deeply explored [12]. In this study, we aim to describe the stress-adaptation mechanism mediated by the *SfHSP70-7* gene of *S. furcifera*. By exploring how the gene responds to temperature and pesticide stress, we attempt to clarify its function in pest pesticide sensitivity, ovarian development, and reproduction. This work will deepen our understanding of the stress resistance of *S. furcifera* and provide a theoretical basis for formulating targeted pest control strategies.

## 2. Materials and Methods

### 2.1. Biological Material

The population of *S. furcifera* used in this study was first collected from the experimental paddy field of the Rice Research Institute of Guizhou Academy of Agricultural Sciences in 2020 and then continuously conserved under laboratory feeding conditions. *S. furcifera* has been continuously maintained on more than 30 generations of healthy rice seedlings under controlled climatic conditions (temperature 25 ± 1 °C, relative humidity 70% ± 5%, 16/8 photoperiod of light/darkness, and no contact with any pesticides). The rice seedlings (*Oryza sativa* L. cv. Xiangliangyou 619) were grown from germinated seeds in plastic pots (12 cm in diameter) under the same controlled conditions, and healthy 15–20-day-old seedlings at the 3–4-leaf stage were used for insect rearing and replaced every 5–7 days. The 3rd-instar nymphs of uniform body size that had newly molted within 24 h were selected for the subsequent experiments.

### 2.2. Test Insecticides and Reagent Preparation

Three commercial insecticides (triflumezopyrim, sulfoxaflor and imidacloprid) were selected as experimental agents [13]. Stock solutions of each insecticide were prepared with analytical-grade acetone (≥99.5%; Sinopharm Chemical Reagent Co., Ltd., Beijing, China), followed by serial gradient dilution using distilled water containing 0.1% Triton X-100 (Sigma-Aldrich, St. Louis, MO, USA) surfactant to form working liquids for bioassay and stress treatment. All insecticide concentrations used in the bioassays and stress treatments are expressed as active ingredient (a.i.) concentrations, which were calculated according to the labeled a.i. content of each commercial formulation (Table S1). Blank solvent (acetone + Triton X-100 aqueous solution) was set as the vehicle control group.

### 2.3. Cloning of SfHSP70-7 Gene

Total RNA was isolated from the mixed developmental stages of *S. furcifera* adults following the instructions of the TransZol Up Kit (TransGen, Beijing, China). RNA integrity and concentration were detected using agarose gel electrophoresis and a spectrophotometer. First-strand cDNA was synthesized using the PrimeScript RT Kit (TaKaRa, Dalian, China) with qualified total RNA as the template. Based on *S. furcifera*’s transcriptome database (SRS27471134), a full-length open reading frame (ORF) primer targeting the *SfHSP70-7* gene was designed (Table S2). PCR amplification products of *SfHSP70* gene purification were obtained using the EasyPure^®^ PCR purification kit (TransGen), inserted into the pGEM-T vector (Promega, Madison, WI, USA), and sequenced by Tsingke Biotechnology (Chongqing, China).

### 2.4. Bioinformatics and Phylogenetic Analysis

ExPASy Proteomics Server predicted the molecular weight, signal peptide, and isoelectric point parameters (http://www.expasy.ch, accessed on 15 June 2026). NCBI BLAST + v2.17.0 tools were used to screen homologous sequences online (https://blast.ncbi.nlm.nih.gov/Blast.cgi, accessed on 14 June 2026), while CLC Sequence Viewer 8 (Qiagen Aarhus A/S, www.qiagenbioinformatics.com, accessed on 15 June 2026) facilitated multi-sequence alignment. Conserved protein domain prediction relied on the SMART webserver (http://smart.embl.de/, accessed on 14 June 2026). The amino acid sequences of HSP70 proteins from 18 insect species were downloaded from the NCBI GenBank database (https://www.ncbi.nlm.nih.gov, accessed on 14 June 2026/). Sequences were selected to represent the major subcellular localization groups of the HSP70 family (cytosolic, ER-resident, and mitochondrial members). Multiple sequence alignment was performed using Clustal W, implemented in MEGA7 with default parameters. No trimming or filtering was performed, resulting in a final alignment of 721 amino acid positions. An NJ-based phylogenetic tree was then constructed in MEGA7 using the Poisson correction substitution model with 1000-bootstrap resampling and pairwise deletion for missing data treatment [14].

### 2.5. Stress Treatment

A two-factor factorial design was employed in the thermal stress bioassay experiment. The first variable contained five temperature levels: 10 °C, 20 °C, 25 °C (untreated ambient control), 30 °C, and 40 °C. The second variable was set to three exposure durations: 10, 30, and 60 min. For each combined treatment, 50 uniformly sized 3rd-instar *S. furcifera* nymphs, confined within one airtight glass vial, served as one biological replicate; four independent biological replicates were prepared. After thermal treatment, all tested nymphs were transferred to the standard feeding environment for a 1 h recovery to allow the induced expression of heat shock genes to fully develop before sampling. Subsequently, insect samples were collected from each treatment group for total RNA extraction for subsequent quantitative PCR detection.

Insecticide exposure assays were conducted using the seedling dipping method [15]. The 3rd-instar nymphs of *S. furcifera* were exposed to LC_50_ concentrations of triflumezopyrim, sulfoxaflor, and imidacloprid (0.18, 2.40, and 0.96 mg/L, respectively) for 6, 12, 24, 36, and 48 h. An aqueous solution containing acetone and Triton X-100 at the same concentration was used as the solvent control group. For each treatment, 80 nymphs with matching sizes served as one biological replicate, and four independent biological replicates were set for all experimental groups. As the insecticide assay was designed as a time-course under continuous exposure, the surviving individuals were sampled directly at each designated time point without a recovery period and collected for total RNA extraction for subsequent qPCR analysis.

### 2.6. Spatiotemporal Expression Profiling of SfHSP70-7 Genes

*S. furcifera* samples were collected from eight different developmental stages under standard rearing conditions without exposure to any temperature or insecticide stressors, including the egg, the 1st to 5th instar nymphs and newly emerged male and female adults. Fifty individual insects were sampled at each developmental stage. For tissue-specific expression profiling, 100 adults were dissected and separated within 24 h after emergence, and nine tissue types were separated, including head, thorax, abdomen, gut, fat body, wings, integument, testes and ovaries. All developmental stages and tissue samples were repeated four times, transferred to 1.5 mL centrifuge tubes without RNase, and stored at −80 °C for total RNA extraction. Following the identical RNA isolation protocol described above, qPCR analysis was performed using the CFX^96^ real-time fluorescence quantitative PCR detection system (Bio-Rad, Hercules, CA, USA). The thermal cycling profile consisted of an initial pre-denaturation step at 94 °C for 3 min, followed by 38 amplification cycles (30 s at 94 °C, 30 s at 60 °C). Melting-curve analysis spanning 60 °C to 95 °C was carried out after cycling completion to confirm specific amplification of single-target amplicons, with four replicates arranged per reaction setup. The *ribosomal protein L9* gene (*SfRPL9*, GenBank accession No. KM885285) and the *α-tubulin* gene (*SfTUB*, GenBank accession No. KP735521) were used as internal reference genes, and the relative transcript abundance of *SfHSP70-7* was quantitatively analyzed using the 2^−ΔΔCT^ method [16].

### 2.7. RNA Interference of SfHSP70-7 Gene

Gene-specific dsRNA primers containing T7 promoter sequence were designed for the target *SfHSP70-7* genes and enhanced green fluorescent protein (*GFP*, negative control). In vitro synthesis of double-stranded RNA (dsRNA) was performed using the TranscriptAid T7 High Yield Transcription Kit (Thermo Fisher Scientific, Waltham, MA, USA), following the manufacturer’s protocols. Purified dsRNA was diluted to a working concentration of 1 μg/μL for subsequent injection use. Prior to microinjection, 3rd-instar nymphs were anaesthetized via brief carbon dioxide exposure inside glass containers. Then, 0.2 μL of the working solution, corresponding to 200 ng of dsRNA, was delivered to the intersegmental thoracic region between the second pair of legs of each nymph using a microinjector from WPI (Worcester, MA, USA), while an equivalent volume (0.2 μL) of ds*GFP* solution at the same concentration was injected as the negative control. Each experimental group contained 100 individuals with four independent biological replicates. At 24 h and 48 h post-injection, 10 surviving nymphs were randomly sampled per group to quantify target gene-silencing efficiency. Surviving individuals were then subjected to insecticide bioassays at the LC_50_ values of the three aforementioned pesticides following the established dipping protocol. Fifty 3rd-instar nymphs with successfully silenced genes were exposed to high-temperature stress treatments at 30 and 35 °C for 10 days, and their survival rates were recorded daily.

An additional cohort of 100 5th-instar nymphs was injected with ds*SfHSP70-7*. Upon adult emergence, males and females from the same treatment were paired for continuous rearing, with oviposition period, number of eggs per female, and egg hatchability systematically recorded throughout the experimental period.

### 2.8. Statistical Analysis

All experimental data were sorted using Excel, and statistical analysis was completed using SPSS 20.0 (IBM, Armonk, NY, USA). Data were analyzed using two-way ANOVA with temperature/insecticide and exposure duration as independent variables, followed by Duncan’s multiple range test (DMRT) for comparisons among all treatment combinations. Differences were considered significant at *p* < 0.05. Significant differences in relative gene expression and corrected mortality among groups were determined using one-way analysis of variance (ANOVA), combined with Duncan’s multiple range test at a significance level of *p* < 0.05. The normality of the data was assessed using the Shapiro–Wilk test, and the homogeneity of variance was assessed using Levene’s test. Graph visualization was completed using GraphPad Prism 9.

## 3. Results

### 3.1. Sequence Analysis of SfHSP70-7 in S. furcifera

**Figure 1:**
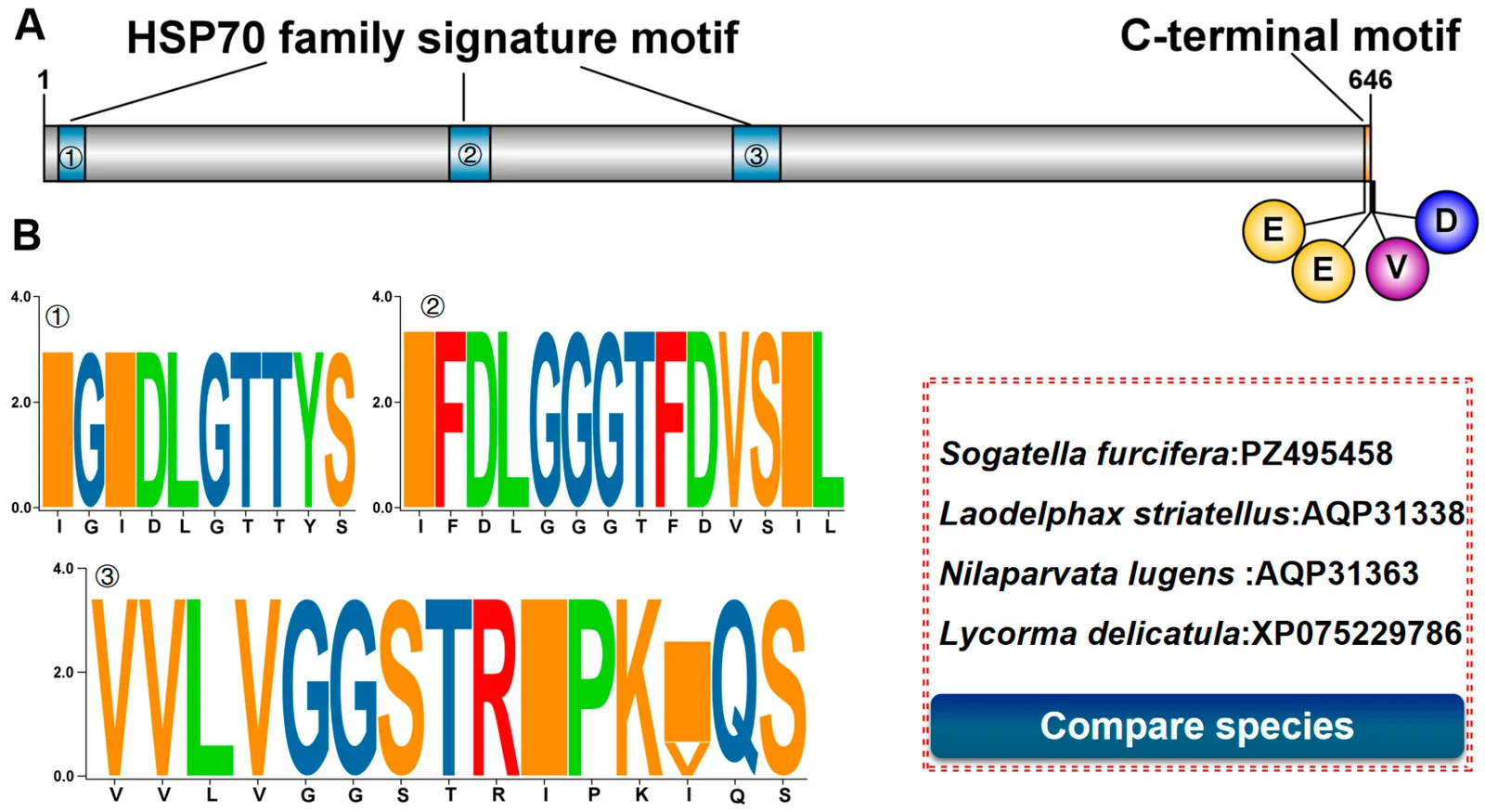
Structural comparison of insect HSP70s. (**A**) Domain structure of SfHSP70-7 predicted by SMART, marking three conserved HSP70 family signature motifs: ① the first conserved signature motif, ② the second conserved signature motif, ③ the third conserved signature motif, as well as the C-terminal motif with EEVD conserved residues. (**B**) Sequence logos of three conserved HSP70 motifs. Aligned hemipteran species and their GenBank accessions are listed on the right.

The complete ORF of *SfHSP70-7* (GenBank accession number PZ495458) spans 1941 bp, encoding a protein with 646 amino acid residues. The predicted molecular weight and isoelectric point (pI) of the SfHSP70-7 protein are 70.86 kD and 5.48, respectively. The functional domain prediction and analysis shows that SfHSP70-7 has three characteristic domains (located at amino acid positions 8–17, 198–211 and 336–350), which are extremely conserved in HSP70 family genes, and contains the cytoplasmic HSP70 conserved sequence “EEVD” at the C-terminal (Figure 1). Multiple sequence alignment analysis demonstrated that SfHSP70-7 shares significant sequence homology with HSP70 proteins from *N. lugens* (Delphacidae; XP_022196826.2), *Lycorma delicatula* (Fulgoridae; XP_075229786.1) and *Corythucha ciliata* (Tingidae; ARW29611.1), with amino acid sequence identities of 93.7%, 84.4%, and 83.9%, respectively.

**Figure 2:**
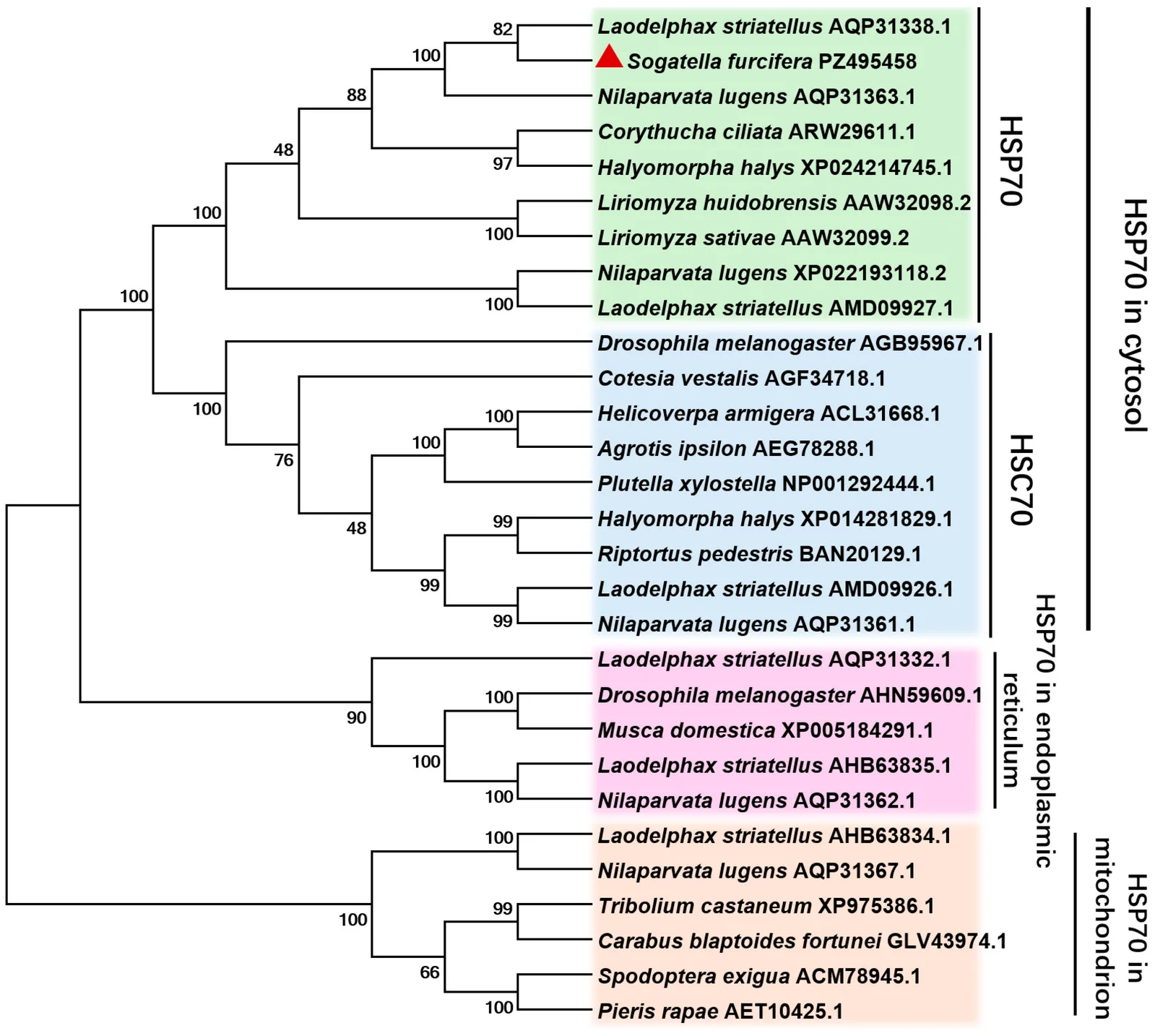
Phylogenetic analysis of insect HSP70s. The unrooted neighbor-joining tree was constructed in MEGA7 based on amino acid sequences, with bootstrap values (1000 replicates) shown at the nodes. GenBank accession numbers are indicated after the species names, and SfHSP70-7 is marked with a red triangle.

Phylogenetic tree analysis shows that the phylogenetic tree contains three main branches: the cytosol HSP70 clade, the endoplasmic reticulum HSP70 clade, and the mitochondrion HSP70 clade (Figure 2). Notably, SfHSP70-7 was classified into the HSP70 branch of the cytosol, which is consistent with the results of the domain analysis. In addition, an evolutionary relationship analysis shows that the SfHSP70-7 of *S. furcifera* and the HSP70 of *L. striatellus* belong to the same evolutionary branch.

### 3.2. Developmental and Tissue-Specific Expression Patterns

**Figure 3:**
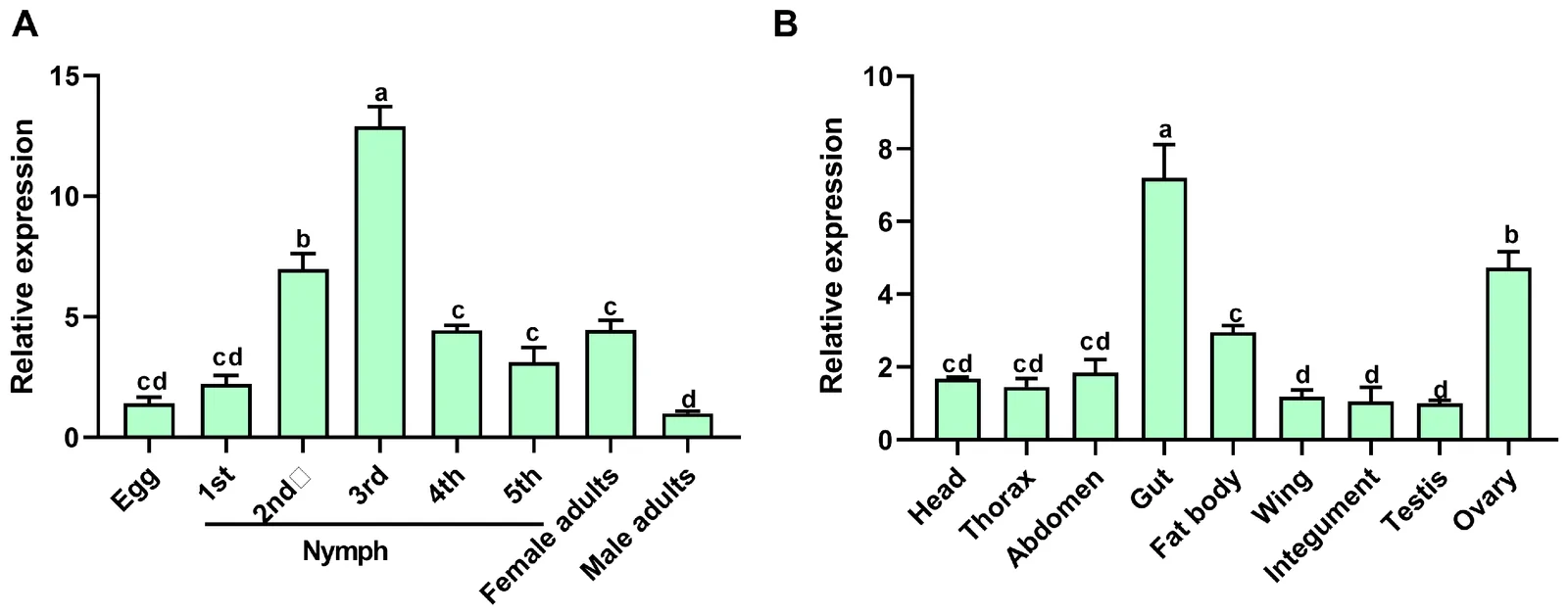
Spatiotemporal expression profiles of *SfHSP70-7* genes in *S. furcifera*. (**A**) Developmental stage-specific expression. (**B**) Tissue-specific expression. Relative expression levels of *SfHSP70-7* were calculated using the 2^−ΔΔCt^ method with *SfRPL9* and *SfTUB* as the internal reference gene. Expression values represent the fold change relative to the male adult (**A**) or the testes (**B**), which was set to 1. One-way ANOVA with Duncan’s multiple range test (DMRT). Different letters denote significant differences at *p* < 0.05.

*SfHSP70-7* was continuously expressed in all developmental stages, and the transcript abundance was the highest in 3rd-instar nymphs. Increased expression levels were also detected in 2nd-instar nymphs and female adults (Figure 3A). *SfHSP70-7* was widely expressed across the anatomical tissues, and its expression levels in the gut and ovaries were particularly high, reaching 7.2 and 4.7 times that in the testes, respectively (Figure 3B).

### 3.3. Expression of SfHSP70-7 Under Different Environmental Stresses

**Figure 4:**
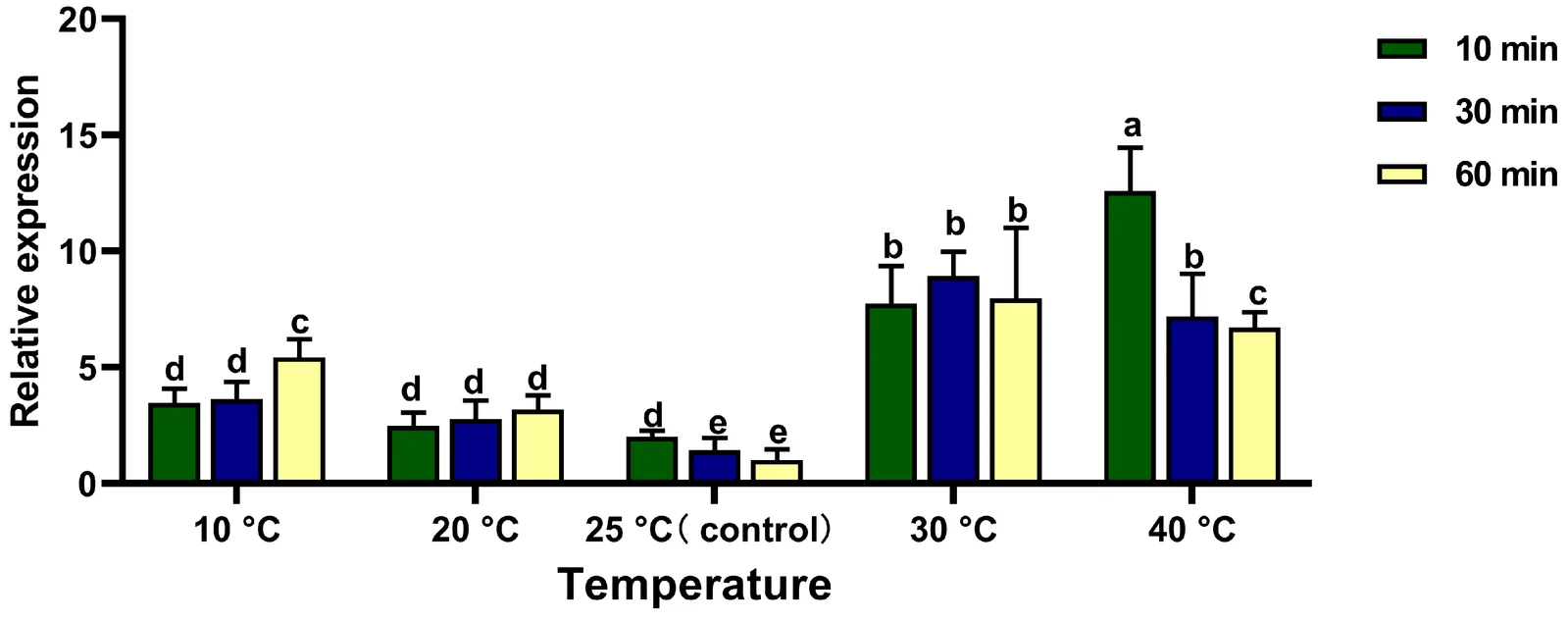
Expression of *SfHSP70-7* in 3rd-instar nymphs of *S. furcifera* exposed to different temperatures (10, 20, 25, 30 and 40 °C) for 10, 30 and 60 min. The 25 °C treatment served as the untreated ambient control. Data were analyzed by two-way ANOVA with temperature and exposure duration as factors, followed by DMRT. Different letters denote significant differences among all treatment combinations at *p* < 0.05.

The expression of *SfHSP70-7* was significantly modulated by both temperature and insecticide stress. Under the temperature treatments, the transcription level was the lowest at 25 °C (the calibration value was 1.0 at 60 min), but it significantly increased at 30 °C and 40 °C. Of these treatments, the highest induced effect (up to 12.5 times) was observed after 10 min at 40 °C (Figure 4). Two-way ANOVA revealed a significant temperature effect (F_4,45_ = 50.65, *p* < 0.001) and a significant temperature × exposure duration interaction (F_8,45_ = 7.20, *p* < 0.001) on *SfHSP70-7* expression, while the main effect of exposure duration was not significant (F_2,45_ = 2.97, *p* = 0.061).

**Figure 5:**
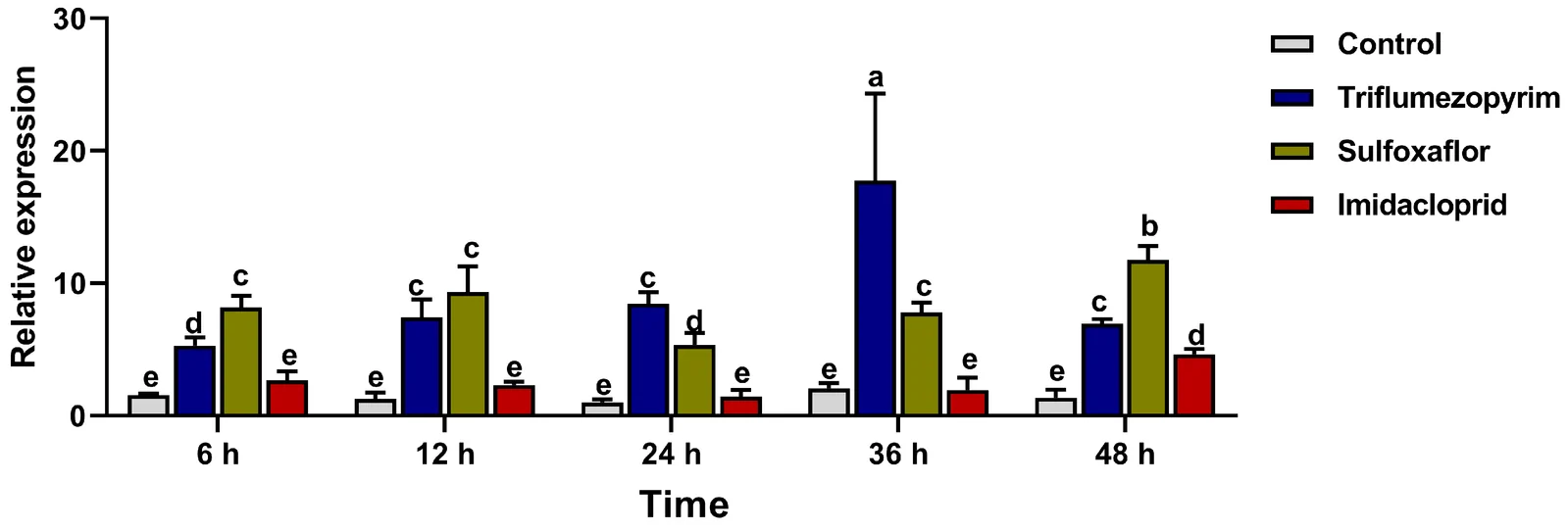
Expression of *SfHSP70-7* in 3rd-instar nymphs of *S. furcifera* exposed to the LC_50_ concentrations of three insecticides (triflumezopyrim, sulfoxaflor and imidacloprid) for 6, 12, 24, 36 and 48 h. Data were analyzed using two-way ANOVA with insecticide treatment and exposure time as factors, followed by DMRT. Different letters denote significant differences at *p* < 0.05.

Under insecticide exposure, all three tested compounds (triflumezopyrim, sulfoxaflor, and imidacloprid) induced higher *SfHSP70-7* expression relative to the untreated control. Triflumezopyrim triggered the strongest response, peaking at 17.7-fold at 36 h, whereas sulfoxaflor reached a maximum of 11.8-fold at 48 h, and imidacloprid caused only moderate upregulation throughout the time course (Figure 5). Two-way ANOVA revealed a significant insecticide treatment effect (*F*_3,60_ = 12.38, *p* < 0.001) and a significant insecticide treatment × exposure time interaction (*F*_12,60_ = 11.43, *p* < 0.001) on *SfHSP70-7* expression, while the main effect of exposure time was not significant (*F*_4,60_ = 0.23, *p* = 0.923).

### 3.4. RNAi-Mediated Knockdown of SfHSP70-7 Increases Insecticide Susceptibility in S. furcifera

**Figure 6:**
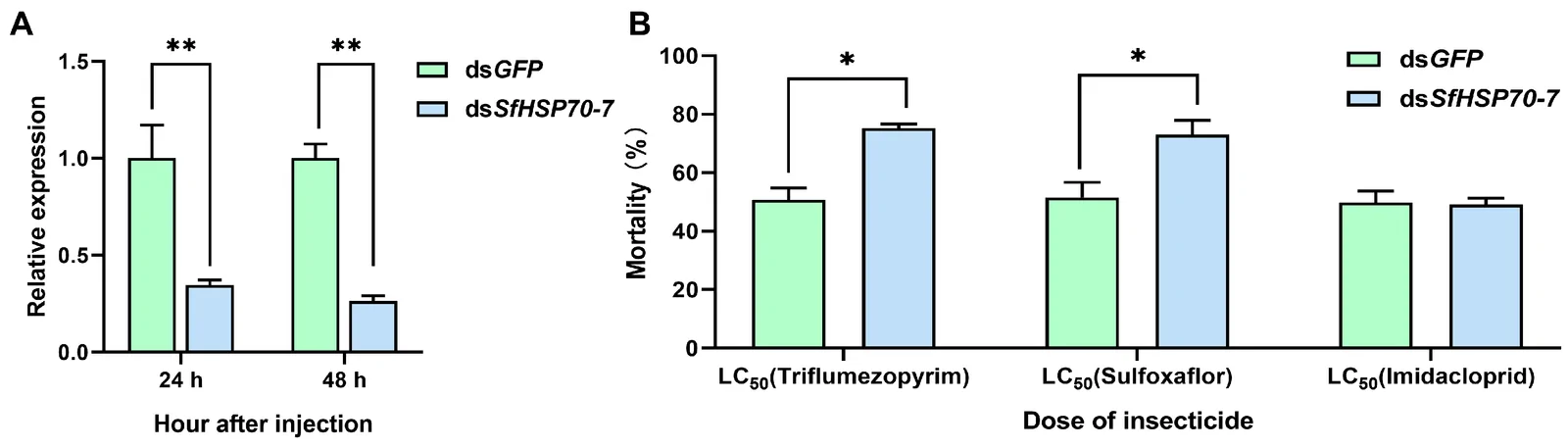
Effects of RNAi targeting *SfHSP70-7* on susceptibility of *S. furcifera* exposed to insecticide. (**A**) Relative expression of *SfHSP70-7* after dsRNA treatment. (**B**) Mortality of 3rd-instar nymphs exposed to LC_50_ concentrations of insecticide following knockdown of *SfHSP70-7*. Significant differences between ds*GFP* and ds*SfHSP70-7*-treated groups were determined using Student’s *t*-test. * *p* < 0.05, ** *p* < 0.01.

RNAi was performed to silence *SfHSP70-7* expression, with ds*GFP* serving as a control. As shown in Figure 6A, injection of ds*SfHSP70-7* significantly reduced the target transcript level by approximately 65.5% and 73.7%, respectively, 24 h and 48 h post-injection compared with the ds*GFP*. Subsequent bioassays revealed that knockdown of *SfHSP70-7* significantly increased the mortality of *S. furcifera* exposed to the LC_50_ concentrations of triflumezopyrim and sulfoxaflor, whereas no significant difference in mortality was observed between the ds*SfHSP70-7* and ds*GFP* groups when treated with imidacloprid (Figure 6B).

### 3.5. Silencing of SfHsp70-7 Reduces Thermotolerance in S. furcifera

**Figure 7:**
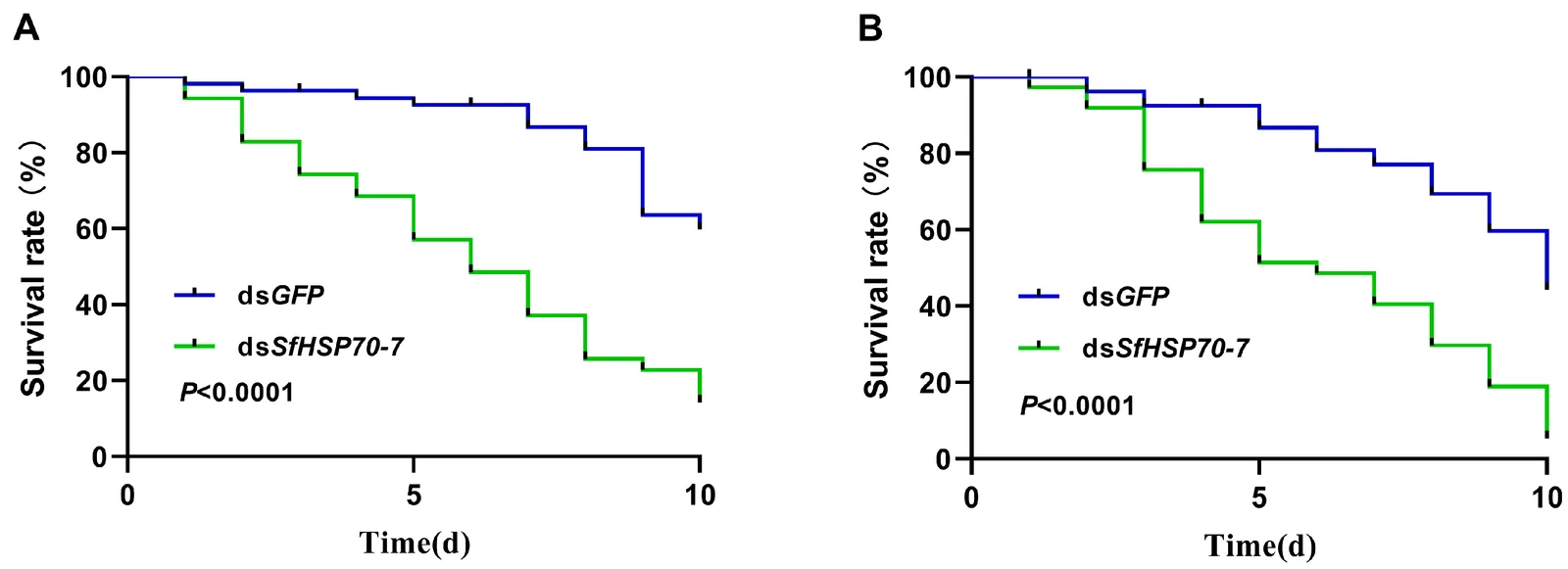
Survival rates of *S. furcifera* after *SfHsp70-7* knockdown under 30 °C (**A**) and 35 °C (**B**) stress. Data were analyzed using Kaplan–Meier survival analysis, and differences were statistically significant (*p* < 0.0001).

After RNAi-mediated silencing of *SfHsp70-7* in *S. furcifera*, insect survival rate under 30 °C and 35 °C decreased significantly relative to the ds*GFP* control group, with reductions of 45.5% and 38.9%, respectively (Figure 7). These results demonstrate that the *SfHsp70-7* gene contributes substantially to high-temperature tolerance in *S. furcifera*.

### 3.6. Knockdown of SfHSP70-7 Impairs Reproductive Performance in S. furcifera

**Figure 8:**
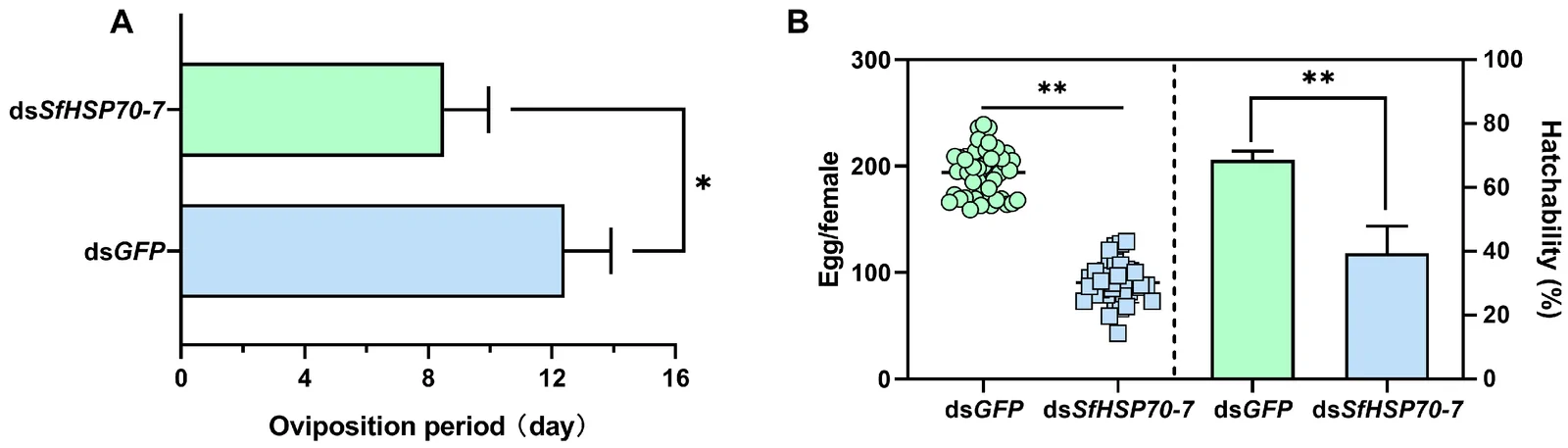
Effect of ds*SfHSP70-7* silencing on fecundity in *S. furcifera*. (**A**) Oviposition period after *SfHSP70-7* RNAi. (**B**) Number of eggs laid per female and hatchability following *SfHSP70-7* knockdown. Data are presented as mean ± SD (*n* = 44 and 26 for oviposition period, n = 48 and 31 for eggs per female, and *n* = 4 per group for hatchability). Significant differences between the ds*GFP* and ds*SfHSP70-7* groups were determined using an unpaired two-tailed Student’s *t*-test after confirming normality (Shapiro–Wilk test) and homogeneity of variance (Levene’s test). * *p* < 0.05, ** *p* < 0.01.

Silencing of *SfHSP70-7* significantly affected key reproductive traits of female *S. furcifera*. As shown in Figure 8A, the oviposition period was notably shortened in the ds*SfHSP70-7*-injected group compared with the ds*GFP* control. Consistently, Figure 8B shows that both the total number of eggs laid per female and hatchability were significantly reduced in the *SfHSP70-7*-silenced individuals.

## 4. Discussion

Extensive research has confirmed that the number of Hsp70 family members, their structural characteristics, and subcellular localization patterns vary considerably across insect species, and this divergence is closely associated with their adaptation to diverse ecological environments [17,18,19,20,21]. In this study, we successfully cloned an *Hsp70* family gene and designated it *SfHSP70-7*. Analysis of the conserved domain architecture revealed that the encoded protein contains the three characteristic signature sequences typical of the Hsp70 family, consistent with the conserved structural features of the Hsp70 superfamily [22]. Notably, the C-terminus of SfHSP70-7 harbors the conserved EEVD motif, a hallmark specific to the cytosolic Hsp70 subfamily [23]. This signature sequence is a critical recognition marker for cytoplasmic Hsp70 proteins, mediating specific interactions with cytosolic co-chaperones and substrate proteins, thereby providing the structural basis for their roles in protein repair and stress regulation within the cytoplasm [24]. Taken together, *SfHSP70-7* exhibits the typical structural characteristics of a cytoplasmic Hsp70 family member, and the high degree of structural conservation suggests that this gene likely participates in insect stress response pathways.

Insect physiological regulation undergoes marked differentiation across developmental stages, and *Hsp70* genes generally exhibit stage-specific expression patterns. In *Plutella xylostella*, *Hsc70* expression remains relatively low in young larvae but becomes significantly upregulated in adults, with higher abundance in females than males [25]. Multiple *Hsp70* genes begin accumulating in *Arma chinensis* from the fifth instar onward, ultimately peaking in the adult stage [26]. Within planthopper groups, multiple *NlHsp70* family members in *N. lugens* show elevated expression in mid-instar nymphs and female adults, a pattern that aligns with feeding and reproductive demands [21]. These observations mirror our finding that *SfHSP70-7* expression peaks significantly in the 3rd-instar nymphs and female adults of *S. furcifera*. Notably, beyond its association with feeding activity, the stage-specific expression pattern of *SfHSP70-7* may also be related to developmental processes. The insect gut undergoes substantial remodeling during development, and 3rd-instar nymphs represent a period of rapid growth with intensive protein synthesis and turnover. Given the strong expression of *SfHSP70-7* in the gut, its upregulation at this stage may contribute to protein homeostasis, cellular remodeling and developmental regulation. In addition, mid-instar nymphs are the stage most frequently exposed to insecticide applications in the field, and the elevated expression at this stage may serve as a proactive defense against such stresses.

Insect Hsp70 also displays pronounced tissue-specific expression. In this study, *SfHSP70-7* showed its highest expression in the gut and ovaries. The gut is the frontline organ confronting rice defensive compounds, whereas the ovaries govern reproductive establishment; both patterns are consistent with the gene’s proposed roles in stress tolerance and reproductive regulation in *S. furcifera*. Comparable findings have been reported in *Ophraella communa* [27], *Monochamus alternatus* [28], *N. lugens* [21], and *Lasioderma serricorne* [29], where Hsp70 enrichment in the midgut and ovaries has been linked to detoxification and oocyte development, respectively. Furthermore, the higher expression in the ovaries than in the testes may be attributed to the intense oogenesis of adult females, including rapid oocyte growth and massive protein synthesis, which imposes a heavier proteostatic burden than spermatogenesis; this is consistent with the severe impairment of female fecundity after *SfHSP70-7* silencing. Similarly, although both the gut and the integument are frontline organs exposed to external stressors, the midgut also serves as the central site of digestion and xenobiotic detoxification with intensive protein turnover and proteotoxic load, whereas the integument functions mainly as a passive physical barrier with lower metabolic activity, suggesting that *SfHSP70-7* expression depends more on the metabolic intensity of a tissue than on exposure alone.

Climate, particularly temperature, is a dominant factor shaping the distribution and population abundance of insects [30]. Cumulative research has confirmed that thermal tolerance in insects is tightly linked to the overexpression of HSP70. In *Agasicles hygrophila*, the transcript levels of *AhHsp70* exhibited a temperature-dependent upward trend under heat shock, with expression intensifying as the thermal stress increased [31]. Heat and cold stress strongly induced the expression of *SmHsp70A1-1* and *SmHsp70A1-2* in *Sitodiplosis mosellana*, yet showed no significant induction when exposed to prolonged extreme temperatures. [32]. A previous study on *S. furcifera* revealed that both high and low temperature treatments significantly stimulated the transcription of all five Hsp70 paralogs [13]. In the present study, treating 3rd-instar nymphs of *S. furcifera* at 10, 20, 30, and 40 °C significantly elevated the expression of *SfHSP70-7*. Collectively, these findings demonstrate that Hsp70 genes occupy a vital position in mediating insect acclimation to variable thermal environments.

As indispensable molecular chaperones, heat shock proteins are readily triggered by insecticides and assist pest populations in developing chemical resistance [1,2]. In *Aphis gossypii*, for instance, sublethal concentrations (LC_25_ and LC_50_) of acetamiprid and sulfoxaflor strongly ramp up the transcript abundance of three *ApHsp70* paralogs after chemical exposure [33]. Low-dose imidacloprid (0.02 and 0.04 ng per female) markedly activates *NlHsp70* expression in *N. lugens*, but this stimulatory effect fades at higher doses, yielding a clear dose-dependent response [8]. A prior study on *S. furcifera* reported an opposite trend: 48 h of exposure to LC_10_ and LC_25_ sublethal doses of thiamethoxam, abamectin, and buprofezin markedly suppressed the transcription of five native *SfHsp70* genes [13]. In *L. striatella*, constitutive *LsHsc70-1/2* were markedly upregulated in chlorpyrifos-resistant strains relative to susceptible ones, yet stress-inducible *LsHsp70-1/2* showed no such inter-strain disparity [7]. Our present data illustrated that LC_50_ treatments of the three tested agrochemicals all boosted the transcription of *SfHSP70-7* in white-backed planthoppers, although imidacloprid yielded a relatively mild induction magnitude. This disparity suggests that individual Hsp70 members carry distinct physiological roles when pests confront insecticide pressure. Future work should leverage whole-genome datasets to dissect the full functional spectrum of the complete Hsp70 repertoire, so as to draw a comprehensive picture of how this gene family mediates insect responses to chemical stress.

RNAi-mediated gene knockdown clearly verifies the dual physiological functions of *SfHSP70-7* in *S. furcifera*. After targeted silencing of this gene, the tolerance of *S. furcifera* to triflumezopyrim and sulfoxaflor was drastically compromised, accompanied by a remarkable increase in mortality. However, insects treated with imidacloprid exhibited no obvious shift in lethal phenotype. Such differentiated responses to distinct insecticides are commonly observed across the Hsp70 families of agricultural pests. Consistent with our findings, RNAi silencing of *ApHsp68* in *A. gossypii* only elevated aphid susceptibility to sulfoxaflor and acetamiprid while exerting negligible regulatory effects on imidacloprid-induced stress [33]. These collective observations indicate that individual Hsp70 paralogs cannot confer broad-spectrum protection against insecticide-induced stress across all insecticide classes. Silencing multiple *TtHsp70* isoforms in *Tetranychus truncatus* drastically reduced mite survival under propargite, abamectin and fenpropathrin stress, yet each member showed distinct protective potency against the three acaricides [34]. Mechanistically, the increased mortality caused by *SfHSP70-7* silencing can be attributed to the canonical chaperone functions of HSP70. Insecticide exposure imposes proteotoxic and oxidative stress on insect cells, as many insecticides stimulate the overproduction of reactive oxygen species (ROS), leading to protein oxidation, misfolding and aggregation [1,2]. As a central guardian of proteostasis, HSP70 binds damaged or misfolded proteins, prevents their aggregation, and facilitates their refolding or degradation, thereby sustaining cellular homeostasis and survival under stress; HSP70 has also been implicated in modulating oxidative stress responses and inhibiting stress-induced apoptosis [35]. Therefore, the induction of *SfHSP70-7* after insecticide exposure can be interpreted as a protective response that buffers insecticide-induced cellular damage, whereas its silencing deprives insects of this chaperone-mediated protection, resulting in the accumulation of cytotoxic protein aggregates and oxidative damage and, ultimately, increased mortality. This interpretation is consistent with previous studies linking HSP70 expression to oxidative stress tolerance and insecticide resistance in other insects [33,34], and the differential effects among these three insecticides may reflect their distinct modes of action and the different degrees of proteotoxic and oxidative stress they impose. Furthermore, loss of functional SfHSP70-7 severely disrupts the full reproductive cascade of female *S. furcifera*. Knockdown of this gene shortened the oviposition period and simultaneously cut down total egg production per female, as well as egg hatching success, demonstrating that this gene acts as an indispensable molecular regulator sustaining ovarian development and offspring reproduction. This conserved reproductive regulatory role of Hsp70 homologs has been validated in multiple insects. Most *NlHsp70* transcripts are highly abundant in the ovaries of the brown planthopper, *N. lugens*, and RNAi silencing blocks vitellin deposition and greatly suppresses population fecundity [26]. Parallel results have been obtained in *O. communa*, where transcriptional repression of *Hsp70* shortened oviposition duration and reduced progeny output [27]. In addition, silencing *LsHSP70-3* in *L. serricorne* drastically impairs ovarian growth, leading to an approximately 70% decline in egg laying and reduced hatchability, which severely undermines pest reproductive fitness [29].

Taken together, *SfHSP70-7* in *S. furcifera* coordinates two core biological processes: it safeguards insects against exogenous chemical stress and modulates female reproductive performance. Its dual involvement in stress resistance and reproduction suggests that this Hsp70 paralog could serve as a potential molecular target for pest control. However, given the high conservation of HSP70 proteins among insects, as revealed by our phylogenetic analysis, potential off-target effects on beneficial and other non-target organisms must be carefully evaluated, and species-specific silencing strategies will be essential before such applications can be realized.

## Data Availability

Data is contained within the article and Supplementary Materials.

## Supplementary Materials

The following supporting information can be downloaded at https://www.mdpi.com/article/10.3390/insects17080765/s1, Table S1: Insecticides information; Table S2. Primers used in this study. Figure S1: Complete coding sequence and predicted amino acid sequence of SfHSP70-7 (PZ495458) in *S. furcifera*.
