# Supplementary material for: Roles of SfHSP70-7 in Thermal Tolerance, Insecticide Adaptability, and Reproductive Performance Under Combined Environmental Stress of the Rice Pest Sogatella furcifera (Hemiptera: Delphacidae)
Source: Insects. 2026 Jul 25;17(8):765. doi: 10.3390/insects17080765 (PMC13513433; doi:10.3390/insects17080765)
Supplement: Supplementary file 1 [file insects-17-00765-s001.zip › insects-4438527-supplementary/Figure S1.pdf]

```
1      ATGCCAGTGAAAACACCAGCGATTGGAATCGATCTCGGAACAACGTACTCGTGTGTCGGAGTGCTTCAACAGGGAAAAGTTGAG
1       M  P  V  K  T  P  A  I  G  I  D  L  G  T  T  Y  S  C  V  G  V  L  Q  Q  G  K  V  E

85     ATTCTAGCCAACGACCAAGGAAACAGGACGACACCCAGCTATGTTGCATTTACGGACTCGGAGCGGCTCATCGGCGATGCGGCC
29      I  L  A  N  D  Q  G  N  R  T  T  P  S  Y  V  A  F  T  D  S  E  R  L  I  G  D  A  A

169    AAAAACCAGGTCGCAATGAATCCGAAAAACACCATCTTCGACGCCAAACGTCTCATCGGTCGCCGTTTCGACGATCCGAAGATC
57      K  N  Q  V  A  M  N  P  K  N  T  I  F  D  A  K  R  L  I  G  R  R  F  D  D  P  K  I

253    ACACAGGACATGAAGCACTGGCCCTTCAAGGTCTACAGCGACTGCGGCAAGCCCAAGATCGAGGTGGACTTCAAGGGCGAGGCG
85      T  Q  D  M  K  H  W  P  F  K  V  Y  S  D  C  G  K  P  K  I  E  V  D  F  K  G  E  A

337    AAGAAGTTTTCTCCTGAGGAAATCAGCGCAATGGTGCTCACGAAAATGAAGGAGACGGCGGAAACCTACCTGGGTGCACCGGTG
113     K  K  F  S  P  E  E  I  S  A  M  V  L  T  K  M  K  E  T  A  E  T  Y  L  G  A  P  V

421    AAGGATGCAGTCATCACCGTTCCAGCCTACTTCAATGACTCACAGAGGCAGGCGACCAAGGATGCTGGAGCTATTGCAGGATTA
141     K  D  A  V  I  T  V  P  A  Y  F  N  D  S  Q  R  Q  A  T  K  D  A  G  A  I  A  G  L

505    AATGTGCTTCGAATTATCAACGAGCCTACAGCTGCTGCCCTTGCCTATGGGTTGGACAAGAACTTGAAAGGAGAGCGCAATGTA
169     N  V  L  R  I  I  N  E  P  T  A  A  A  L  A  Y  G  L  D  K  N  L  K  G  E  R  N  V

589    CTCATTTTCGACCTGGGAGGAGGTACATTCGATGTCTCAATCCTAACCATCGACGAGGGCTCTCTATTTGAGGTGAGATCTACA
197     L  I  F  D  L  G  G  G  T  F  D  V  S  I  L  T  I  D  E  G  S  L  F  E  V  R  S  T

673    GCAGGAGACACTCATCTGGGAGGAGAGGATTTTGACAACCGGCTTGTGGATCATCTATGTGAAGAATTCAAGAGGAAATATCGT
225     A  G  D  T  H  L  G  G  E  D  F  D  N  R  L  V  D  H  L  C  E  E  F  K  R  K  Y  R

757    AAAGACATCAAGTCCAATCCACGAGCTTTGAGAAGATTGAGAACTGCAGCTGAGAGGGCAAAGAGAACACTCTCTTCCAGCACT
253     K  D  I  K  S  N  P  R  A  L  R  R  L  R  T  A  A  E  R  A  K  R  T  L  S  S  S  T

841    GAGGCCAGCATTGAAATAGATGCACTGTGTGAAGGAGTCGACTTCTACACCAAAGTATCACGAGCACGATTTGAAGAATTGTGC
281     E  A  S  I  E  I  D  A  L  C  E  G  V  D  F  Y  T  K  V  S  R  A  R  F  E  E  L  C

925    GCAGATCTATTCCGTTCCACACTGCATCCAGTTGAGAAAGCCCTGAACGACGCCAAGATGGACAAATCATCAATCCATGATGTT
309     A  D  L  F  R  S  T  L  H  P  V  E  K  A  L  N  D  A  K  M  D  K  S  S  I  H  D  V

1009   GTACTTGTTGGAGGATCCACTCGTATACCCAAGATACAATCACTATTGCAGAACTTCTTCAATGGAAAATCACTCAACTTTTCA
337     V  L  V  G  G  S  T  R  I  P  K  I  Q  S  L  L  Q  N  F  F  N  G  K  S  L  N  F  S

1093   ATCAACCCAGACGAAGCTGTCGCTTATGGAGCAGCCGTGCAAGCAGCTATACTCAGTGGAGATCAGAGCTCACAAATCCAGGAC
365     I  N  P  D  E  A  V  A  Y  G  A  A  V  Q  A  A  I  L  S  G  D  Q  S  S  Q  I  Q  D

1177   GTTCTTCTAGTCGATGTGACACCTCTCTCACTGGGCATTGAAACTGCAGGGGGAGTGATGGCGAAAATAATTGAACGCAACAGC
393     V  L  L  V  D  V  T  P  L  S  L  G  I  E  T  A  G  G  V  M  A  K  I  I  E  R  N  S

1261   AGAATTCCATGCAAGCAGTCACAAACCTTCAGCACTTATGCAGATAACCAGCCAGCTGTTACAATCCAAGTATTTGAAGGTGAG
421     R  I  P  C  K  Q  S  Q  T  F  S  T  Y  A  D  N  Q  P  A  V  T  I  Q  V  F  E  G  E

1345   AGGGCGATGACTAAGGACAACAATCTACTGGGAACGTTTGATTTGACTGGAATACCACCAGCACCTAGAGGAGTACCGAAAATT
449     R  A  M  T  K  D  N  N  L  L  G  T  F  D  L  T  G  I  P  P  A  P  R  G  V  P  K  I

1429   GAGGTCACGTTTGATTTGGATGCAAACGGTATTTTGAATGTGTCTGCTAAGGAGACTGGAACTGGAATCACAAAGAATATTGTG
477     E  V  T  F  D  L  D  A  N  G  I  L  N  V  S  A  K  E  T  G  T  G  I  T  K  N  I  V

1513   ATCAAAAATGATAAGGGACGTCTGTCGAAAGCTGATATTGATAGAATGGTGAACGAAGCTGAACAGTTTAAGGAGGAGGACGAA
505     I  K  N  D  K  G  R  L  S  K  A  D  I  D  R  M  V  N  E  A  E  Q  F  K  E  E  D  E

1597   AAACAGAAGCAGAGAGTGAGCGCAAGAAATCAGCTGGAAAACTACATCTTCAGTGTGAAACAAGCGCTGGATGAAGCAGGAGGG
533     K  Q  K  Q  R  V  S  A  R  N  Q  L  E  N  Y  I  F  S  V  K  Q  A  L  D  E  A  G  G

1681   AAACTGAGTGACGGTGATAAGTCGGAGGTGAAACAGAGATGCGACGAGTGCTTGCAGTGGCTTGATAACAACACACTCGCCGAT
561     K  L  S  D  G  D  K  S  E  V  K  Q  R  C  D  E  C  L  Q  W  L  D  N  N  T  L  A  D

1765   AAGGAGGAGTTCGAGCACAAGCTGAAGGAGCTGCAGCAGCTATGCTCACCAATCATGGCCAAGATCTACTCGAGTGGAGCAGCT
589     K  E  E  F  E  H  K  L  K  E  L  Q  Q  L  C  S  P  I  M  A  K  I  Y  S  S  G  A  A

1849   GGAACTGGTGCTCAACCCACTTCCTGTGGACAACAGTTCAGCCAGGGCCAGTCATTCCCACGAAATGGACCTACTGTCGAGGAG
617     G  T  G  A  Q  P  T  S  C  G  Q  Q  F  S  Q  G  Q  S  F  P  R  N  G  P  T  V  E  E

1933   GTAGATTAA
645     V  D  *
```

Figure S1. Complete coding sequence and predicted amino acid sequence of *SfHSP70-7* (PZ495458) in *S. furcifera*. Sequence alignment and annotation were performed with DNAMAN 6.0 software. The upper line shows the nucleotide sequence, and the lower line indicates the corresponding translated protein sequence.
