# Supplementary material for: Roles of SfHSP70-7 in Thermal Tolerance, Insecticide Adaptability, and Reproductive Performance Under Combined Environmental Stress of the Rice Pest Sogatella furcifera (Hemiptera: Delphacidae)
Source: Insects. 2026 Jul 25;17(8):765. doi: 10.3390/insects17080765 (PMC13513433; doi:10.3390/insects17080765)
Supplement: Supplementary file 1 [file insects-17-00765-s001.zip › insects-4438527-supplementary/Supplementary table.pdf]

Table S1 Insecticides information

Active ingredient	Formulation	Manufacturing company	Pesticide registration certificate number	LC_{50} (mg a.i./L)	Equivalent formulation conc. (mg/L)
Triflumezopyrim	10% SC	Kedihua agriculture technology co., ltd	PD20171740	0.18	1.8
Sulfoxaflor	22% SC	Kedihua agriculture technology co., ltd	PD20160336	2.40	10.91
Imidacloprid	25% SP	Jiangsu kesheng group co., ltd	PD2010258	0.96	3.84

Table S2. Primers used in this study

Experiments	Gene name	Primer Sequence (5′ to 3′)
ORF confirmation	*SfHSP70-7*	F: ACCAGCGATTGGAATCGATCTC<br>R: TAATCTACCTCCTCGACAGT
qPCR analysis	*SfHSP70-7*	F: AGAACTGCAGCTGAGAGGGC<br>R: GGATTGATGATTTGTCCATC
	SfTUB	F: CGCTGTTGATGGAGAGGCTGTC<br>R: ACGACGGCTGTGGATACCTGTG
	SfRPL9	F: GTGAACAAGTGCGAAGGA<br>R: TCATAGCAGTGCGTCAAC
dsRNA synthesis	*SfHSP70-7*	F:TAATACGACTCACTATAGGGTGTGGATCATC TATGTGAAGA<br>R:TAATACGACTCACTATAGGGATAAGCGACAG CTTCGTCTGG
	GFP	F: TAATACGACTCACTATAGGGGGTTGAT CACGTCGCAAGCTA<br>R: TAATACGACTCACTATAGGGGAGGTTT CCCTCTGGCTTGTT

F: forward primer; R: reverse primer
